# Efficacy of pharmacologic therapies for reducing bronchopulmonary dysplasia and mortality in preterm infants: a network meta-analysis

**DOI:** 10.1186/s12887-026-07408-y

**Published:** 2026-07-28

**Authors:** Runjia Zhao, Wei Guo

**Affiliations:** 1 West China School of Pharmacy, Sichuan University, Chengdu, China; 2 School of Pharmacy, Hebei Medical University, Shijiazhuang, 050017 China

**Keywords:** Bronchopulmonary dysplasia, Pharmacological therapy, Preterm infants, Prevention

## Abstract

**Background:**

Bronchopulmonary dysplasia (BPD) is a major complication of prematurity and is associated with substantial respiratory morbidity, impaired growth, and increased mortality in severe cases. Pharmacologic prevention strategies have been evaluated in randomized trials, but their comparative effects remain uncertain.

**Aim:**

This network meta-analysis aimed to summarize the comparative evidence for pharmacologic interventions evaluated for reducing BPD incidence and pre-discharge mortality in preterm infants.

**Methods:**

We searched PubMed, Embase, Cochrane Library, and Web of Science for randomized controlled trials (RCTs) investigating pharmacological therapies for BPD prevention, with a search cutoff of September 1, 2025. Data analysis was conducted using R 4.2.3 software. The primary outcome was BPD incidence, and the secondary outcome was pre-discharge mortality. BPD definitions and assessment timepoints were extracted from individual studies and interpreted considering variation across NIH 2001, NIH 2018, Shennan, or study-specific criteria.

**Results:**

Thirty-six randomized controlled trials involving 11,414 preterm infants were included in the systematic review. Of these, 24 trials contributed to the BPD incidence network, and 19 trials contributed to the pre-discharge mortality network. Several interventions showed comparative signals for lower odds of BPD incidence or pre-discharge mortality when compared with placebo; however, the strength and precision of these signals varied across comparisons. In the BPD incidence network, some estimates excluded the null, but several comparisons were informed partly by indirect evidence or sparsely connected nodes. In the pre-discharge mortality network, favorable estimates were less certain because mortality events were sparse and several 95% credible intervals (CrIs) were wide. The certainty of evidence was moderate for BPD incidence and low for pre-discharge mortality. SUCRA rankings were therefore interpreted only as probabilistic summaries within the network and not as evidence of clinical superiority.

**Conclusion:**

This network meta-analysis identified several comparative signals for pharmacologic prevention of BPD and reduction of pre-discharge mortality in preterm infants. However, these findings are constrained by low-to-moderate certainty of evidence, indirect comparisons, sparse network nodes, and imprecision in several estimates. The results should therefore be interpreted as evidence-mapping and hypothesis-generating rather than as treatment recommendations. Future studies should prioritize direct head-to-head comparisons, phenotype- or severity-specific analyses, and long-term respiratory and neurodevelopmental outcomes.

**Supplementary Information:**

The online version contains supplementary material available at 10.1186/s12887-026-07408-y.

## Background

Bronchopulmonary dysplasia (BPD) is the most common chronic lung disease in preterm infants, particularly those born at a gestational age (GA) of less than 32 weeks or with a birth weight (BW) under 1,500 g [1, 2]. Reported incidence estimates for bronchopulmonary dysplasia vary substantially depending on the diagnostic definition used, the timing of assessment, and the population studied. In very preterm infants, BPD is commonly assessed at 36 weeks’ postmenstrual age using NIH-based or related diagnostic frameworks, and reported incidence may range widely across clinical settings. Rates are generally higher among infants born at lower gestational ages and birth weights, particularly in extremely preterm populations [3]. The etiology of BPD is primarily associated with premature birth, mechanical ventilation, oxygen toxicity, and infection, all of which contribute to impaired lung development and abnormal tissue repair [4].

At present, the prevention and treatment of BPD have become a major clinical concern [5] and the main clinical treatments at this stage include nutritional support, fluid intake restriction, and medications, including glucocorticoids (GCs), surfactants, caffeine, vitamin A, macrolide antibiotics, nitric oxide (NO), diuretics, and monoclonal antibody drugs [6–8]. GCs are commonly used in clinical practice. Although GCs have been evaluated for reducing the risk of BPD, but uncertainty remains regarding optimal timing, dose, route, safety, target population, and long-term outcomes [9]. Further research is needed to clarify the optimal timing, dose, route of administration, safety profile, and long-term outcomes of these therapies [10]. Currently, there is a lack of consensus concerning whether vitamin A is capable of reducing the BPD incidence, and more studies are needed for further evaluation [11]. Macrolide antibiotics may lower the risk of BPD in preterm babies, especially in those with Ureaplasma urealyticum infections; however, they are not recommended for routine use [12]. In BPD prevention and treatment, the role of inhaled nitric oxide in BPD prevention remains controversial [13, 14]. The appropriate use of diuretics may improve short-term pulmonary mechanics in selected infants, but evidence for a definitive reduction in BPD incidence remains limited; however, attention needs to be paid to the side effects of long-term use [15]. Importantly, most studies have compared these drugs with placebo or standard care rather than directly with each other, which limits clinicians’ ability to determine the most effective and safest option. Currently, there is a lack of direct head-to-head comparisons between pharmacological therapies for the prevention of BPD.

Traditional pairwise meta-analyses are limited in their ability to compare multiple interventions that have often been evaluated against placebo or standard care rather than directly against each other. Network meta-analysis provides a framework for synthesizing both direct and indirect evidence across available interventions, while allowing uncertainty and network structure to be considered. Therefore, this network meta-analysis aimed to summarize comparative evidence across pharmacologic interventions evaluated for reducing BPD incidence and pre-discharge mortality in preterm infants. Rather than establishing treatment recommendations, the purpose was to explore relative comparative effects, characterize the uncertainty of the current evidence base, and identify priorities for future head-to-head and phenotype-specific research.

## Materials and methods

This network meta-analysis was prospectively registered in PROSPERO (CRD42023443297) and conducted in accordance with the Preferred Reporting Items for Systematic Reviews and Meta-Analyses (PRISMA) guidelines [16].

### Search strategy

We systematically searched PubMed, Embase, Cochrane, and Web of Science databases for randomized controlled trials (RCTs) assessing pharmacological prevention of BPD, with the search deadline set as September 1, 2025. The search strategy was carefully developed by combining medical subject heading (MeSH) terms and relevant free-text terms such as ‘bronchopulmonary dysplasia’, ‘prevention’, ‘reduction’, and ‘control’. The full search strategy is outlined in Supplementary Material 1. No language restrictions were applied. clinical trial registries (ClinicalTrials.gov) and grey literature sources were screened to minimize publication bias.

### Inclusion and exclusion criteria

Studies were included if they met the following criteria:

Population: Very preterm infants (GA < 32 weeks) and/or very low birth weight (< 1500 g) [17]. Where reported, we extracted detailed GA ranges to describe the population characteristics more precisely.

Interventions: Pharmacologic therapies evaluated for the prevention of BPD or reduction of BPD-related pulmonary morbidity in preterm infants were eligible. To improve clinical interpretability, interventions were organized into clinically coherent classes, including corticosteroids, surfactant-based therapies, methylxanthines, vitamin supplementation, macrolides, nitric oxide-related therapies, antioxidant or anti-inflammatory agents, and emerging biologic or cell-based therapies. We acknowledge that these interventions differ in mechanism of action, clinical indication, timing of administration, feasibility, and context of use. Therefore, they were not assumed to be clinically interchangeable in routine practice. Rather, they were considered comparable within a broad preventive framework because they were all studied in preterm infants at risk of BPD and were intended to reduce lung injury, inflammation, impaired alveolar development, or related downstream complications. To assess the plausibility of the transitivity assumption, we extracted and reviewed major potential effect modifiers across studies, including gestational age, birth weight, baseline respiratory support, exposure to mechanical ventilation, infection or inflammatory status where reported, treatment timing, and important co-interventions. Nevertheless, given the heterogeneity of these factors, particularly for indication-specific therapies, all indirect comparisons were interpreted cautiously.

Comparators: Placebo or standard care with no additional pharmacological BPD-preventive therapy.

Primary Outcome: The primary outcome was BPD incidence, and the secondary outcome was pre-discharge mortality. For BPD incidence, we extracted the diagnostic definition and assessment timepoint used in each trial, including NIH 2001, NIH 2018, Shennan 1988, or study-specific criteria where applicable. Because death before discharge may preclude subsequent ascertainment of BPD, composite outcomes such as “BPD or death” were recorded descriptively when reported. However, BPD incidence and pre-discharge mortality were analyzed as separate prespecified outcomes in the primary network meta-analysis. Composite outcomes were not used as the principal pooled outcome unless explicitly stated. For the primary BPD incidence network, trials using NIH 2001/2018 or comparable oxygen-based BPD definitions assessed around 36 weeks’ postmenstrual age were included. Trials using Shennan 1988 or other non-comparable study-specific definitions were retained for sensitivity analyses to examine the robustness of the findings across alternative BPD definitions.

Study Design: Randomized controlled trials (RCTs).

The exclusion criteria included duplicate literature, animal studies, case reports, conference abstracts, reviews, inaccessible full texts, and studies on other organic diseases.

### Data extraction

Based upon predefined inclusion and exclusion criteria, two authors rigorously screened the literature. All disagreements were handled by discussing or seeking the input of a third party for consultation and reached a consensus. Information extracted from the included studies included the following key details: first author, year of publication, geographic location, sample size, male sex, GA, BW, definitions and diagnosis of BPD, interventions, and outcomes.

### Handling of missing outcome data

Outcome availability was assessed separately for each prespecified outcome. Studies meeting the overall eligibility criteria were retained in the systematic review even if one or more outcomes of interest were not reported. However, for a given outcome-specific network meta-analysis, studies were included only when sufficient numerical data for that outcome were available from the published report or could be reliably derived.

When outcome data were incomplete or not directly extractable, attempts were made to contact the corresponding authors for clarification where feasible. If no response was obtained and the relevant outcome data could not be reliably extracted, the study was excluded from that specific quantitative synthesis but remained part of the overall evidence base of the review.

To improve transparency, we recorded outcome-specific availability and reasons for non-inclusion in each network. These reasons included outcome not reported, outcome reported without extractable numerical data.

### Risk of bias assessment

Risk of bias was assessed using the revised Cochrane risk-of-bias tool for randomized trials (RoB 2.0) [18]. The following domains were evaluated: bias arising from the randomization process, bias due to deviations from intended interventions, bias due to missing outcome data, bias in outcome measurement, and bias in selection of the reported result. Each trial was judged as having low risk of bias, some concerns, or high risk of bias. Two reviewers independently performed the assessment, and disagreements were resolved through discussion or consultation with a third reviewer.

The risk-of-bias assessment was not used to assign statistical weights or to exclude studies from the primary network meta-analysis. Instead, it was used descriptively to characterize the methodological quality of the included trials, to inform GRADE downgrading for the risk-of-bias domain, and to guide the cautious interpretation of treatment estimates, particularly when favorable signals were driven by studies with some concerns or high risk of bias.

### Grade of evidence

The certainty of evidence was assessed using the GRADE [19] framework adapted for network meta-analysis. Evidence from randomized controlled trials was initially rated as high certainty and could be downgraded for risk of bias, inconsistency, indirectness, imprecision, and publication bias. For network estimates, incoherence between direct and indirect evidence was also considered when relevant. The risk-of-bias domain was informed by the RoB 2.0 assessments of the included trials contributing to each outcome. Inconsistency was judged using model-fit comparison, node-splitting results, and the overlap of direct and indirect evidence where available. Indirectness was assessed according to population, intervention, comparator, outcome definition, treatment timing, and the transitivity assumption. Imprecision was judged according to the width of the 95% credible intervals and whether the intervals crossed the null or included clinically important benefit and harm. Publication bias was assessed using comparison-adjusted funnel plots and Egger’s test when applicable.

The certainty ratings were used to guide interpretation rather than to modify the statistical model. Therefore, treatment rankings and individual drug signals were interpreted considering the corresponding certainty of evidence, with caution applied to sparse treatment nodes, indirect comparisons, and estimates with wide 95% credible intervals (CrIs). The certainty ratings were assessed at the outcome/network level rather than for each individual treatment comparison. Therefore, the certainty ratings of “moderate” for BPD incidence and “low” for pre-discharge mortality should be interpreted as overall certainty judgments for the corresponding outcome networks.

### Data analysis

A Bayesian random-effects network meta-analysis [20] was conducted to compare the efficacy of different pharmacologic interventions for preventing BPD incidence and pre-discharge mortality in preterm infants. Binary outcomes were summarized as odds ratios (ORs) with corresponding 95% CrIs. The analysis was performed in R (version 4.2.3) using the gemtc package.

A consistency model was used for the primary network meta-analysis. Model fit was further explored by comparison with an inconsistency model using the deviance information criterion (DIC). Lower DIC values indicate better model fit, and differences greater than 5 points were considered potentially meaningful; however, DIC was interpreted as an index of relative model fit rather than as a formal statistical test of inconsistency.

For the Bayesian model, we used the default weakly informative priors implemented in the gemtc package for treatment effects and between-study heterogeneity. Four Markov chain Monte Carlo (MCMC) chains were run, each with 2,000 iterations, including 1,000 burn-in iterations, yielding 4,000 post-burn-in samples in total. Convergence was assessed using the Gelman–Rubin diagnostic, with the potential scale reduction factor (PSRF) examined for all model parameters, together with visual inspection of trace plots and review of effective sample sizes. PSRF values close to 1.00 were considered indicative of acceptable convergence. Effective sample sizes were also reviewed to ensure adequate posterior sampling stability, specific prior distributions, zero-event handling, continuity correction procedures, PSRF ranges, and effective sample sizes are provided in the Supplementary Methods. Multi-arm trials were incorporated directly into the network model while preserving the within-study correlation among treatment arms. For sparse binary data and studies with zero events in one or more arms, estimation was performed within the Bayesian framework supported by the network model; where required for computational stability, continuity correction procedures were applied consistently [21]. Local inconsistency was assessed using node-splitting methods by comparing direct and indirect estimates for comparisons with available closed loops. A P value < 0.05 was considered suggestive of statistical inconsistency. When evidence of inconsistency or important heterogeneity was suspected, sensitivity analyses were undertaken to examine the robustness of the findings. All statistical analyses were performed using R software (version 4.2.3).

## Results

**Fig. 1:**
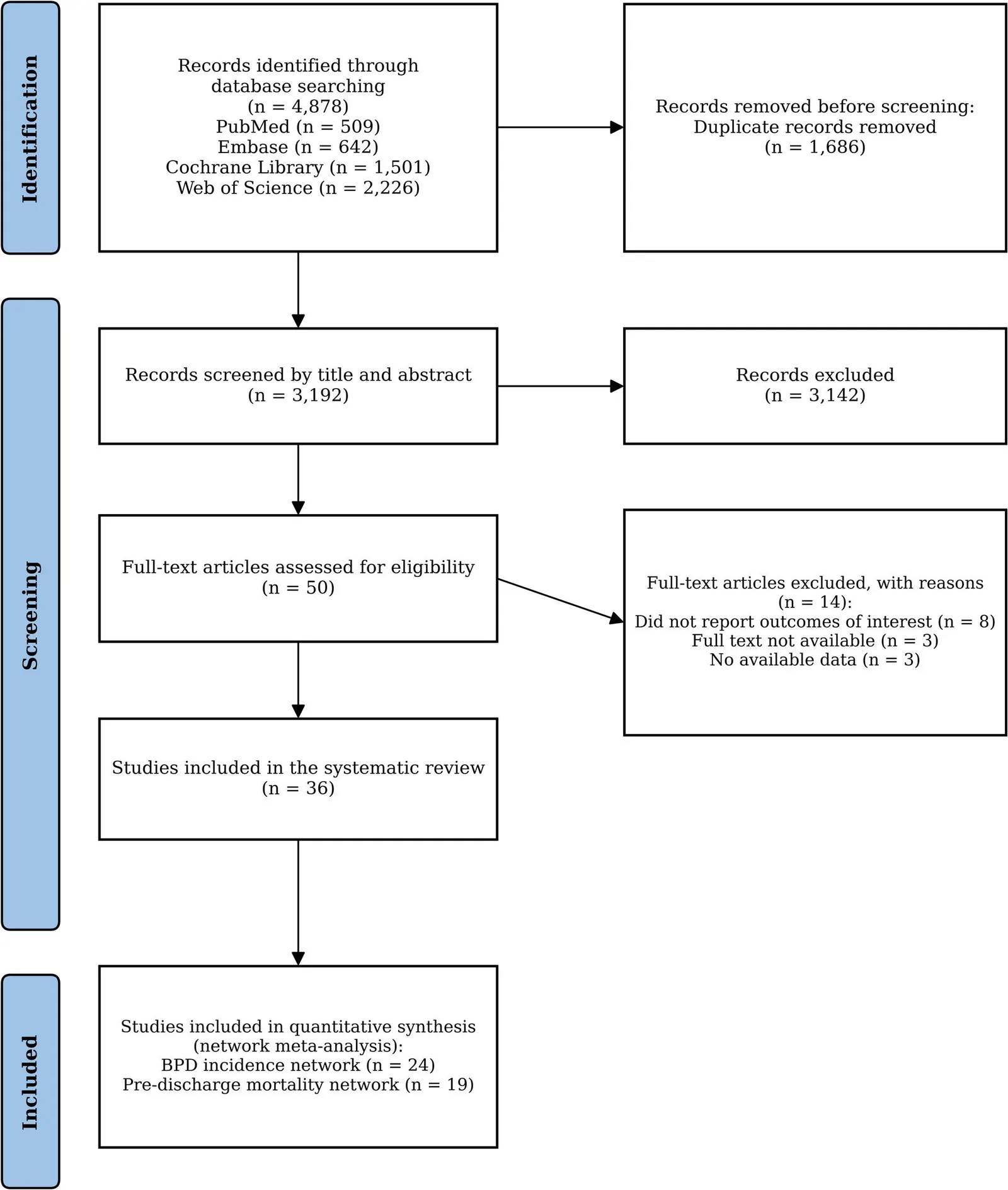
PRISMA flow diagram of the study process. PRISMA, Preferred Reporting Items for

A total of 4,878 records were identified through searches of PubMed (*n* = 509), Embase (*n* = 642), the Cochrane Library (*n* = 1,501), and Web of Science (*n* = 2,226). After removal of 1,686 duplicate records, 3,192 records were screened by title and abstract. Of these, 3,142 records were excluded, and 50 full-text articles were assessed for eligibility. Fourteen full-text articles were excluded with reasons, leaving 36 RCTs [22–57] involving 11,414 neonates included in the systematic review (Table 1; Fig. 1). Contribution to the quantitative synthesis differed by outcome because not all trials reported both BPD incidence and pre-discharge mortality in an extractable format. Specifically, 24 trials contributed to the network meta-analysis of BPD incidence, and 19 trials contributed to the network meta-analysis of pre-discharge mortality. A detailed outcome-level accounting of study contribution and reasons for non-inclusion is provided (Supplementary Material 2 Table S1). The included interventions were reported using standard generic names, including allopurinol, indomethacin, salbutamol, beclomethasone, dexamethasone, N-acetylcysteine, recombinant human CuZn superoxide dismutase, nitric oxide, azithromycin, lucinactant, erythromycin, vitamin A, hydrocortisone, budesonide, fluticasone propionate, ibuprofen, sildenafil, UCB-derived stem cells, poractant alfa, vitamin D, nitric oxide plus vitamin A, zinc, caffeine, and placebo.

**Table 1:** Characteristics of the included studies

Study	Country/region	Sample size	Male sex	BPD diagnostic criteria	Assessment timepoint	Clinical intent	Outcome priority	Number of BPD cases	Gestational age, weeks	Birth weight, g	Intervention regimen	Outcomes
Russell 1995 [51]	England	allopurinol: 205placebo: 195	allopurinol: 54placebo: 48	Shennan 1988 definition	Early postnatal	Prophylactic	Primary	allopurinol: 48placebo: 48	allopurinol: 29placebo: 29	allopurinol: 1240placebo: 1298	20 mg/kg, enteral (orogastric tube), once daily, 7 days	F1; F2
Couser 1996 [32]	USA	indomethacin: 43placebo: 47	indomethacin: 25placebo: 22	Shennan 1988 definition	Within 24 h of birth	Prophylactic	Primary	indomethacin: 28placebo: 23	indomethacin: 26.4placebo: 26.4	indomethacin: 915placebo: 879	Indomethacin 0.1 mg/kg, IV infusion, every 24 h, 6 doses	F1; F2
Denjean 1998 [36]	France	salbutamol: 44beclomethasone: 43placebo: 43	/	Shennan 1988 definition	Day 10 of life	Treatment of evolving BPD	Primary	salbutamol: 24beclomethasone: 18placebo: 37	salbutamol: 27.7beclomethasone: 27.6placebo: 27.8	salbutamol: 1056beclomethasone: 1060placebo: 1082	Salbutamol 200 µg/day, inhalation, every 4 h, 28 days with 8-day taper + Beclomethasone 1000 µg/day, inhalation, 4× daily, 28 days with 8-day taper	F1; F2
Cole 1999 [31]	England	beclomethasone: 123placebo: 130	beclomethasone: 51placebo: 53	Shennan 1988 definition	3–14 days of life	Prophylactic	Primary	beclomethasone: 19placebo: 23	beclomethasone: 26placebo: 26	beclomethasone: 800placebo: 802	beclomethasone: inhalation5 µg-40 µg/kg, once daily, 28 days	F1;
Nupponen 2002 [49]	Finland	dexamethasone: 15placebo: 15	dexamethasone: 8placebo: 11	Shennan 1988 definition	Day 1 of life	Ultra-early anti-inflammatory	Primary	dexamethasone: 1placebo: 7	dexamethasone: 29.1placebo: 29.2	dexamethasone: 1223placebo: 1250	dexamethasone: inhalation0.25-0.5 mg/kg, once daily, 4 days	F1; F2
Ahola 2003 [24]	Finland	N-acetylcysteine: 194placebo: 197	N-acetylcysteine: 95placebo: 99	Shennan 1988 definition	<36 h of life	Prophylactic	Primary	N-acetylcysteine: 64placebo: 68	N-acetylcysteine: 26.3placebo: 26.5	N-acetylcysteine: 776placebo: 780	N-acetylcysteine: IV16 to 32 mg/kg/d, 6 days	F1; F2
Davis 2003 [35]	USA	recombinant human CuZn superoxide dismutase: 154placebo: 148	recombinant human CuZn superoxide dismutase: 49placebo: 49	NIH 2001 definition	At birth	Prophylactic	Primary	recombinant human CuZn superoxide dismutase: 36placebo: 37	recombinant human CuZn superoxide dismutase: 27placebo: 27	recombinant human CuZn superoxide dismutase: 873placebo: 875	recombinant human CuZn superoxide dismutase: inhalation5 mg/kg, once daily, 28 days	F1; F2
Ballard 2006 [28]	USA	nitric oxide: 294placebo: 288	nitric oxide: 115placebo: 162	NIH 2001 definition	7–21 days of life	Treatment	Primary	nitric oxide: 149placebo: 164	nitric oxide: 26placebo: 26	nitric oxide: 766placebo: 759	nitric oxide: inhalation20 ppm, once daily, 24 days	F1; F2
Dani 2006 [33]	Italy	nitric oxide: 20placebo: 20	/	Shennan 1988 definition	≤7 days of life	Prophylactic	Primary	nitric oxide: 6placebo: 12	nitric oxide: 26.3placebo: 26.7	nitric oxide: 937placebo: 825	nitric oxide: inhalation10 ppm, once daily, 12 days	F1; F2
Doyle 2006 [37]	Australia	dexamethasone: 35placebo: 35	dexamethasone: 16placebo: 21	Shennan 1988 definition	≤7 days of life	Treatment	Primary	dexamethasone: 28placebo: 29	dexamethasone: 24placebo: 25	dexamethasone: 652placebo: 700	dexamethasone: inhalation0.89 mg/kg, once daily, 10 days	F1; F2
Kinsella 2006 [44]	USA	nitric oxide: 398placebo: 395	nitric oxide: 211placebo: 216	Shennan 1988 definition	Early postnatal	Treatment	Secondary	nitric oxide: 212placebo: 210	nitric oxide: 25.6placebo: 25.6	nitric oxide: 796placebo: 788	nitric oxide: inhalation5 ppm, once daily, 21 days	F1; F2
Ballard 2007 [26]	USA	azithromycin: 19placebo: 16	azithromycin: 7placebo: 8	Shennan 1988 definition	≤7 days of life	Prophylactic	Secondary	azithromycin: 9placebo: 10	azithromycin: 25.6placebo: 25.3	azithromycin: 762placebo: 736	azithromycin: IV10 mg/kg, once daily, 7 days	F1; F2
Laµghon 2009 [45]	USA	lucinactant 90 mg/kg: 47lucinactant 175 mg/kg: 45placebo: 44	lucinactant 90 mg/kg: 30lucinactant 175 mg/kg: 24placebo: 17	Shennan 1988 definition	Early postnatal	Treatment	Secondary	lucinactant 90 mg/kg: 23lucinactant 175 mg/kg: 21placebo: 22	lucinactant 90 mg/kg: 25.5lucinactant 175 mg/kg: 26placebo: 25.7	lucinactant 90 mg/kg: 733lucinactant 175 mg/kg: 773placebo: 753	lucinactant 90 mg/kg: inhalation90 mg/kg, once daily, 12 dayslucinactant 175 mg/kg: inhalation175 mg/kg once daily, 12 days	F1; F2
Ballard 2011 [27]	USA	azithromycin: 111placebo: 109	azithromycin: 62placebo: 50	Shennan 1988 definition	Early postnatal	Prophylactic	Secondary	azithromycin: 69placebo: 71	azithromycin: 25.7placebo: 26	azithromycin: 803placebo: 810	azithromycin: IV10 mg/kg, once daily, 7 days	F1; F2
Gharehbaghi 2012 [40]	Iran	azithromycin: 56placebo: 52	azithromycin: 34placebo: 35	NIH 2001 definition	3–14 days of life	Prophylactic	Secondary	azithromycin: 3placebo: 9	azithromycin: 29.8placebo: 29.7	azithromycin: 1177placebo: 1212	azithromycin: oral10 mg/kg, once daily, 7 days	F1
Ng 2012 [48]	China	erythromycin: 19placebo: 26	erythromycin: 9placebo: 12	/	Day 1 of life	Treatment	Primary	erythromycin: 4placebo: 9	erythromycin: 28.4placebo: 28.4	erythromycin: 1114.8placebo: 1179.5	erythromycin: oral5 mg/k once daily, 14 days	F1
Terrin 2013 [54]	Italy	zinc: 97placebo: 96	zinc: 37placebo: 43	/	<36 h of life	Prophylactic	Primary	zinc: 10placebo: 15	zinc: 28placebo: 28	zinc: 1114placebo: 1033	zinc: oral9 mg, once daily, 14 days	F1
Gadhia 2014 [38]	USA	nitric oxide: 253nitric oxide plus vitamin A: 118vitamin A: 111placebo: 253	nitric oxide: 46nitric oxide plus vitamin A: 47vitamin A: 41placebo: 49	NIH 2001 definition	At birth	Treatment	Primary	nitric oxide: 144nitric oxide plus vitamin A: 67vitamin A: 65placebo: 145	nitric oxide: 25.74nitric oxide plus vitamin A: 25.65vitamin A: 26.86placebo: 26.3	nitric oxide: 800nitric oxide plus vitamin A: 810vitamin A: 810placebo: 790	nitric oxide: 5 ppm, inhalation, once daily, 14 days.vitamin A: NR	F1; F2
Kiatchoosakun 2014 [43]	Thailand	vitamin A: 40placebo: 40	vitamin A: 20placebo: 21	NIH 2001 definition	7–21 days of life	Treatment	Secondary	vitamin A: 20placebo: 21	vitamin A: 29placebo: 28.9	vitamin A: 1152.8placebo: 1121.3	vitamin A: 5, 000 IU, once daily, 24 days.;	F1; F2
Bassler 2015 [29]	Switzerland	budesonide: 437placebo: 419	budesonide: 222placebo: 213	NIH 2001 definition	≤7 days of life	Prophylactic	Secondary	budesonide: 101placebo: 138	budesonide: 26.1placebo: 26.1	budesonide: 798placebo: 803	budesonide: inhalation0.25 mg/kg, once daily, 14 days	F1; F2
Baud 2016 [30]	France	hydrocortisone: 255placebo: 266	hydrocortisone: 131placebo: 149	NIH 2001 definition	≤7 days of life	Treatment	Secondary	hydrocortisone: 50placebo: 70	hydrocortisone: 26.4placebo: 26.5	hydrocortisone: 860placebo: 840	hydrocortisone: inhalation1 mg/kg, once daily, 7 days	F1; F2
Nakamura 2016 [47]	Japan	fluticasone propionate: 107placebo: 104	fluticasone propionate: 63placebo: 50	NIH 2001 definition	3–14 days of life	Prophylactic	Secondary	fluticasone propionate: 38placebo: 43	fluticasone propionate: 26.1placebo: 26.2	fluticasone propionate: 783.87placebo: 784.06	fluticasone propionate: inhalation4.2 mg once daily, 28 days	F1; F2
Yeh 2016 [57]	China	budesonide: 131placebo: 134	budesonide: 71placebo: 72	NIH 2001 definition	Day 1 of life	Prophylactic	Secondary	budesonide: 38placebo: 67	budesonide: 26.5placebo: 26.8	budesonide: 882placebo: 935	budesonide: inhalation0.25 mg/kg, once daily, 14 days	F1; F2
Hasan 2017 [42]	USA	nitric oxide: 229placebo: 222	nitric oxide: 115placebo: 116	NIH 2001 definition	<36 h of life	Treatment	Primary	nitric oxide: 130placebo: 137	nitric oxide: 25.6placebo: 25.6	nitric oxide: 750placebo: 724	nitric oxide: inhalation5 ppm, twice daily, 21 days	F1; F2
Lin 2017 [46]	China	indomethacin: 73ibuprofen: 71	indomethacin: 38ibuprofen: 35	NIH 2001 definition	At birth	Prophylactic	Primary	indomethacin: 51ibuprofen: 45	indomethacin: 26.3ibuprofen: 26.2	indomethacin: 812ibuprofen: 801	indomethacin: IV, 0.2 mg/kg, twice daily, 21 daysibuprofen: IV, 10 mg/kg, twice daily, 21 days	F1; F2
Abounahia 2019 [22]	Qatar	sildenafil: 20placebo: 20	sildenafil: 13placebo: 11	NIH 2001 definition	7–21 days of life	Treatment	Primary	sildenafil: 3placebo: 3	sildenafil: 26.9placebo: 26.7	sildenafil: 1089.1placebo: 972.5	sildenafil: oral0.5 mg/kg, twice daily, 21 days	F1; F2
Onland 2019 [50]	Netherlands	hydrocortisone: 182placebo: 190	hydrocortisone: 96placebo: 109	NIH 2001 definition	≤7 days of life	Treatment	Secondary	hydrocortisone: 100placebo: 95	hydrocortisone: 25.4placebo: 25.6	hydrocortisone: 775placebo: 710	hydrocortisone: inhalation5 mg/kg, twice daily, 7 days	F1; F2
Giridhar 2020 [41]	India	vitamin A: 61placebo: 59	vitamin A: 36placebo: 32	NIH 2001 definition	≤7 days of life	Prophylactic	Secondary	vitamin A: 5placebo: 7	vitamin A: 31placebo: 31	vitamin A: 1047placebo: 1084	vitamin A: 15, 000 IU, twice daily, 7 days	F1; F2
Sung 2020 [53]	Korea	ibuprofen: 70placebo: 72	ibuprofen: 28placebo: 41	NIH 2001 definition	3–14 days of life	Treatment	Secondary	ibuprofen: 29placebo: 27	ibuprofen: 26.8placebo: 26.7	ibuprofen: 893placebo: 915	ibuprofen: oral10 mg/kg, twice daily, 7 days	F1; F2
Ahn 2021 [23]	Korea	UCB-derived stem cells: 33placebo: 33	UCB-derived stem cells: 24placebo: 17	NIH 2001 definition	Day 1 of life	Prophylactic	Secondary	UCB-derived stem cells: 16placebo: 18	UCB-derived stem cells: 25.2placebo: 25.2	UCB-derived stem cells: 755placebo: 740	UCB-derived stem cells: inhalation1 × 10^7 cells/kg, twice daily, 7 days	F1; F2
Dargaville 2021 [34]	Australia	poractant alfa: 241placebo: 244	poractant alfa: 125placebo: 119	NIH 2018 definition	<36 h of life	Prophylactic	Secondary	poractant alfa: 81placebo: 102	poractant alfa: 27.3placebo: 27.3	poractant alfa: 929placebo: 928	poractant alfa: inhalation200 mg/kg, twice daily, 7 days	F1; F2
Gharehbaghi 2021 [39]	Iran	budesonide: 64placebo: 64	budesonide: 38placebo: 40	NIH 2018 definition	At birth	Treatment	Primary	budesonide: 24placebo: 38	budesonide: 28.2placebo: 28.4	budesonide: 1055placebo: 1089	budesonide: inhalation0.25 mg/kg, twice daily, 7 days	F1; F2
Rysavy 2021	USA	vitamin A: 405placebo: 402	vitamin A: 202placebo: 196	NIH 2018 definition	7–21 days of life	Prophylactic	Primary	vitamin A: 5placebo: 7	vitamin A: 25.8placebo: 25.8	vitamin A: 770placebo: 769	vitamin A: 15, 000 IU, twice daily, 7 days	F1; F2
Yao 2021 [56]	China	budesonide: 47placebo: 47	budesonide: 26placebo: 28	NIH 2018 definition	≤7 days of life	Prophylactic	Primary	budesonide: 5placebo: 7	budesonide: 30.7placebo: 30.5	budesonide: 1325.63placebo: 1350.48	budesonide: inhalation0.5 mg/kg, twice daily, 14 days	F1
Aristizabal 2023 [25]	USA	vitamin D 200 IU: 19vitamin D 800 IU: 23placebo: 31	vitamin D 200 IU: 12vitamin D 800 IU: 11placebo: 14	NIH 2018 definition	≤7 days of life	Prophylactic	Primary	vitamin D: 5placebo: 7	vitamin D 200 IU: 26vitamin D 800 IU: 25placebo: 25	vitamin D 200 IU: 846vitamin D 800 IU: 839placebo: 773	vitamin D: oral200-800 IU, twice daily, 14 days	F1
Schmidt 2006 [52]	Australia	Caffeine: 1006placebo: 1000	/	NIH 2001 definition	≤7 days of life	Prophylactic	Primary	caffeine: 5placebo: 7	/	/	Caffeine: IV 20 mg, twice daily, 14 days	F1; F2

*BPD* bronchopulmonary dysplasia, *GA*, gestational age, *BW* birth weight, *IV* intravenous, *IM* intramuscular, *NR* not reported, *F1* BPD incidence, *F2* pre-discharge mortality, *NIH* National Institutes of Health, *UCB* umbilical cord blood. All pharmacologic interventions are reported using standard generic names. Doses are presented as dose, route, frequency, and treatment duration where available. “/” indicates not reported

The methodological quality of the included trials varied. Most trials were judged as having low risk of bias in the randomization process, whereas some studies raised concerns related to deviations from intended interventions, incomplete adherence reporting, or missing outcome data. The RoB 2.0 assessment (Supplementary Material 2 Figure S1) was used to inform the risk-of-bias domain of the GRADE assessment (Supplementary Material 2 Table S2) and to guide interpretation of the network estimates. It was not used as a study-exclusion criterion or as a statistical weighting factor in the primary network meta-analysis.

The certainty of evidence was rated as moderate for BPD incidence and low for pre-discharge mortality. For BPD incidence, certainty was downgraded mainly because of risk-of-bias concerns and imprecision in some treatment comparisons, particularly those involving sparsely connected nodes. For pre-discharge mortality, certainty was downgraded because of risk-of-bias concerns, imprecision, and limited direct evidence for several comparisons. Therefore, although several interventions showed favorable comparative signals, these findings should be interpreted according to the corresponding certainty of evidence rather than by SUCRA rankings alone.

### Results of consistency modeling

The current study used a random-effects model to compare the difference in DIC between consistent and inconsistent modeling, and the absolute value of the difference in DIC was < 5, The results (Supplementary Material 2 Table S3) suggested comparable model fit, DIC differences were interpreted cautiously and not considered definitive evidence of consistency. Node-splitting analyses revealed no statistically significant differences between direct and indirect estimates for any comparison (all *P* > 0.10). Given the relatively sparse nature of some treatment nodes, the absence of statistically significant inconsistency should be interpreted cautiously.

### Primary analysis (NIH 2001/2018)

#### Primary outcome (BPD incidence)

**Fig. 2:**
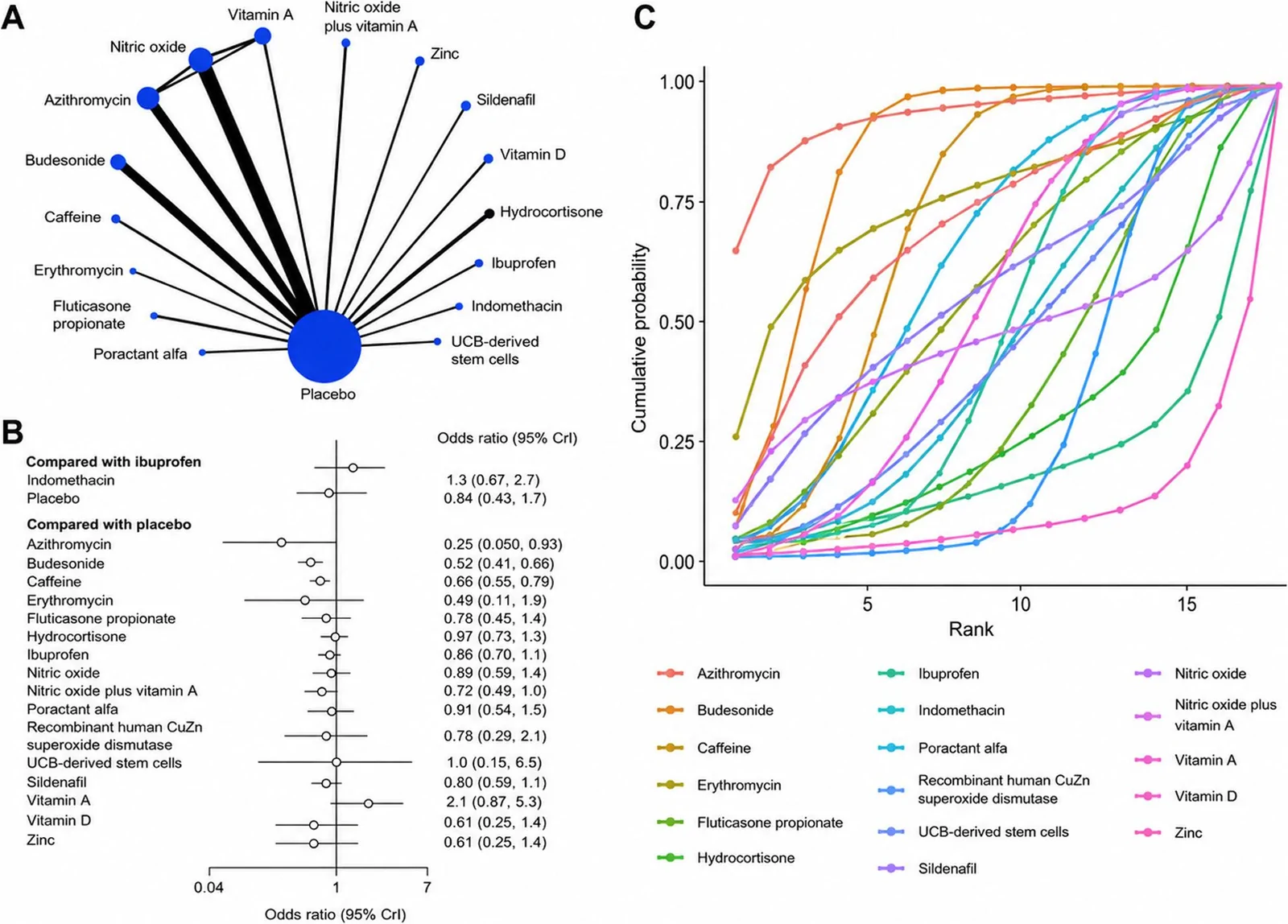
Network meta-analysis of BPD incidence. (**A**) Network plot of pharmacologic interventions for BPD incidence. Each node represents one intervention, and each edge represents a direct comparison between interventions. Node size is proportional to the number of participants assigned to each intervention, and edge thickness is proportional to the number of direct comparisons. (**B**) Forest plot of network estimates versus placebo. Effect estimates are presented as odds ratios (ORs) with 95% credible intervals (CrIs). (**C**) SUCRA-based ranking plot for BPD incidence. Higher SUCRA values indicate a higher probability of favorable ranking within the network, but rankings should be interpreted together with effect estimates, CrIs, network structure, and certainty of evidence.For graphical readability, shortened labels may be used in the figure only; all treatment names are defined using standard generic names. BPD, bronchopulmonary dysplasia; OR, odds ratio; CrI, credible interval; SUCRA, surface under the cumulative ranking curve

Twenty-four trials involving 8,032 neonates contributed to the network meta-analysis of BPD incidence (Fig. 2). A closed loop was identified in the network, and node-splitting analysis for the VA versus NO comparison showed no statistically significant evidence of inconsistency between the direct and indirect estimates (Supplementary Material 2, Figure S2). Compared with placebo, azithromycin [OR = 0.25, 95% CrI: 0.05–0.93], budesonide [OR = 0.52, 95% CrI: 0.41–0.66], and caffeine [OR = 0.66, 95% CrI: 0.55–0.79] were associated with lower odds of BPD incidence (Fig. 2B). In addition, azithromycin was associated with lower odds of BPD than hydrocortisone [OR = 0.26, 95% CrI: 0.05–0.98], ibuprofen [OR = 0.21, 95% CrI: 0.04–0.93], and vitamin D [OR = 0.12, 95% CrI: 0.02–0.59] (Supplementary Material 2, Table S4A).

According to SUCRA, azithromycin ranked highest (0.93), followed by budesonide (0.85) and erythromycin (0.74), whereas vitamin D ranked lowest (0.07) (Fig. 2C and Supplementary Material 2, Table S5). Part of the apparent comparative advantage may have been informed by indirect evidence within the network, and several estimates—particularly for sparsely connected treatment nodes—were accompanied by wide CrIs. Although azithromycin, budesonide, and caffeine showed favorable estimates compared with placebo, these signals should be interpreted within the overall moderate-certainty BPD incidence network and should not be understood as comparison-specific GRADE judgments. Estimates involving sparsely connected nodes or wide 95% CrIs should not be interpreted as definitive evidence of clinical superiority.

#### Secondary outcome (pre-discharge mortality)

**Fig. 3:**
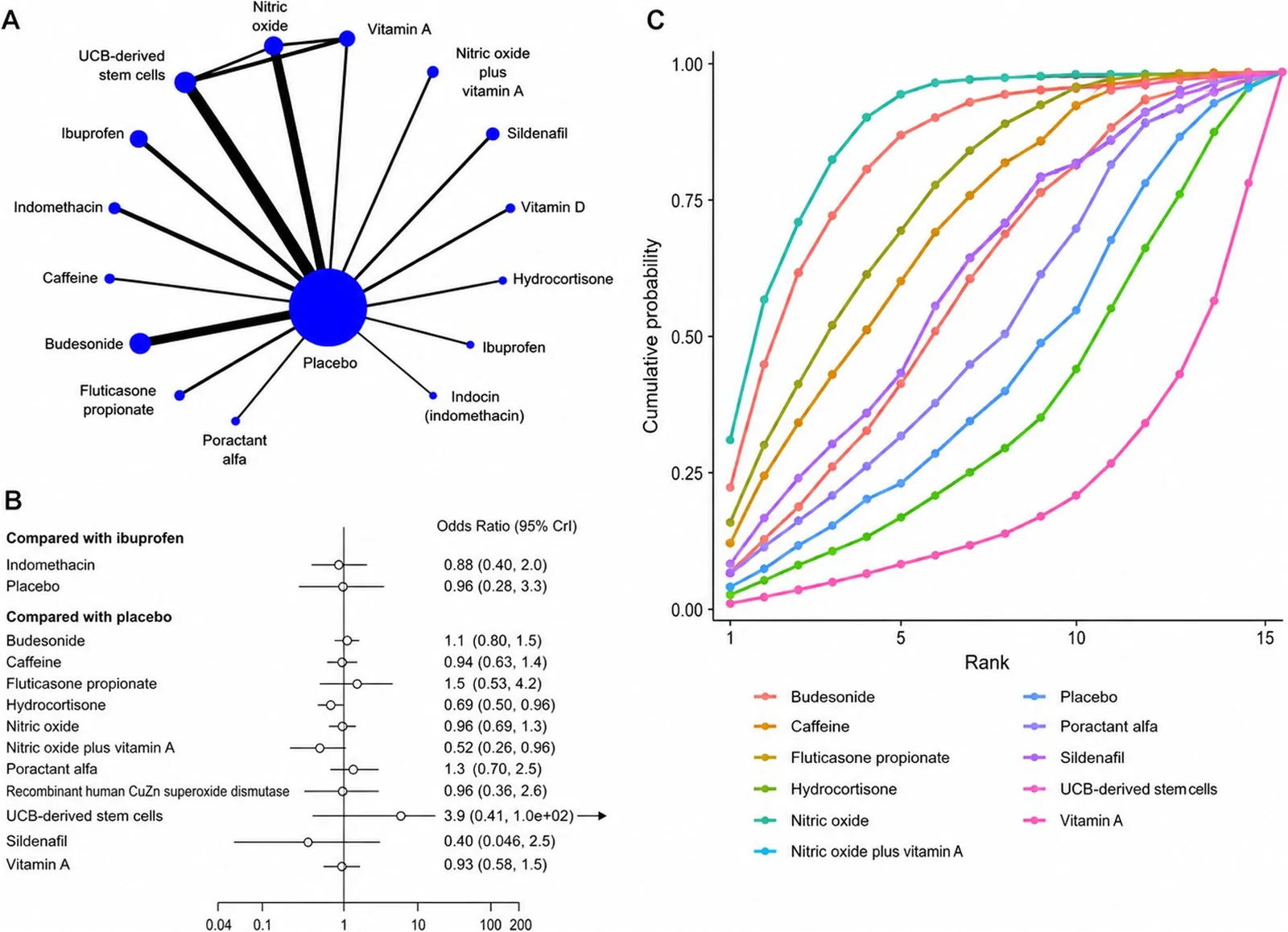
Network meta-analysis of pre-discharge mortality. (**A**) Network plot of pharmacologic interventions for pre-discharge mortality. Each node represents one intervention, and each edge represents a direct comparison between interventions. Node size is proportional to the number of participants assigned to each intervention, and edge thickness is proportional to the number of direct comparisons. (**B**) Forest plot of network estimates versus placebo. Effect estimates are presented as odds ratios (ORs) with 95% credible intervals (CrIs). (**C**) SUCRA-based ranking plot for pre-discharge mortality. Higher SUCRA values indicate a higher probability of favorable ranking within the network, but rankings should not be interpreted as definitive evidence of clinical superiority. For graphical readability, shortened labels may be used in the figure only; all treatment names are defined using standard generic names. OR, odds ratio; CrI, credible interval; SUCRA, surface under the cumulative ranking curve

Nineteen trials involving 7,669 neonates contributed to the network meta-analysis of pre-discharge mortality (Fig. 3). A closed loop was also identified in this network, and node-splitting analysis for the VA versus NO comparison showed no statistically significant evidence of inconsistency between the direct and indirect estimates (Supplementary Material 2, Figure S3). Compared with placebo, hydrocortisone [OR = 0.69, 95% CrI: 0.50–0.96] and nitric oxide plus vitamin A [OR = 0.52, 95% CrI: 0.26–0.96] were associated with lower odds of pre-discharge mortality (Fig. 1B). In addition, nitric oxide plus vitamin A was associated with lower odds of pre-discharge mortality than budesonide [OR = 0.47, 95% CrI: 0.23–0.95] (Supplementary Material 2, Table S4B).

According to SUCRA, nitric oxide plus vitamin A ranked highest (0.87), followed by sildenafil (0.81) and hydrocortisone (0.77), whereas UCB-derived stem cells ranked lowest (0.13) (Fig. 1C and Supplementary Material 2, Table S5). However, these findings should be interpreted with caution. The mortality network contained limited direct evidence for several comparisons, some treatment nodes were sparse, and precision was limited for part of the network. Accordingly, some of the apparent comparative advantage may have been driven partly by indirect comparisons. Although hydrocortisone and nitric oxide plus vitamin A showed favorable estimates compared with placebo, the certainty of evidence for pre-discharge mortality was low. Therefore, these findings should be interpreted as cautious comparative signals rather than definitive evidence of mortality benefit.

### Publication bias

For outcomes with enough studies, comparison-adjusted funnel plots were constructed to explore potential small-study effects within the network meta-analysis framework. Egger’s regression test was performed as an exploratory assessment where applicable.

**Fig. 4:**
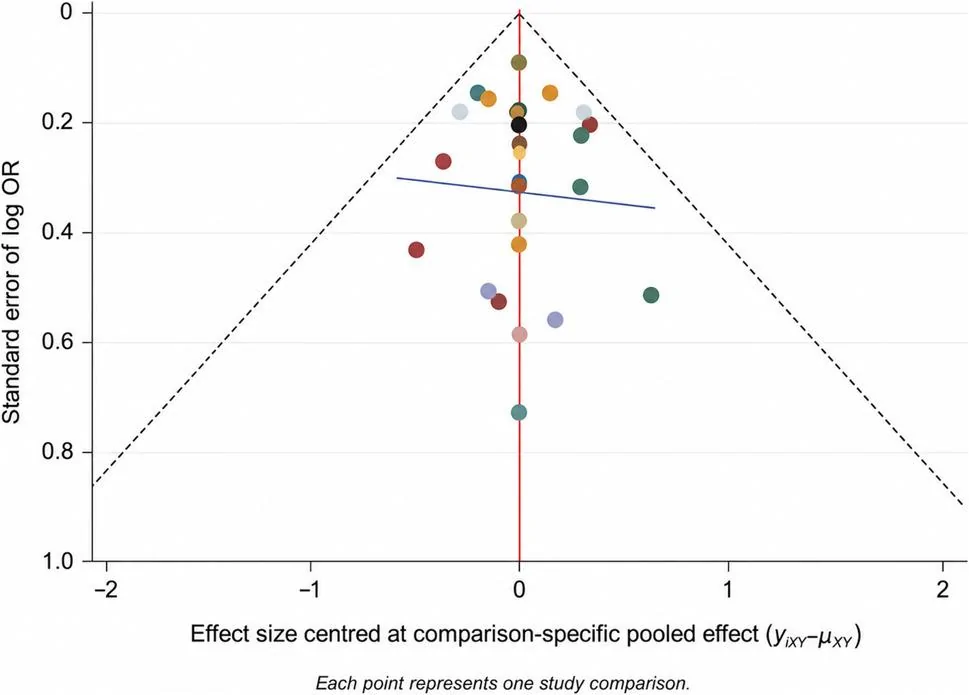
Comparison-adjusted funnel plot for BPD incidence. Each point represents one study comparison centered at the comparison-specific pooled effect on the log OR scale. The vertical red line indicates the null-centered comparison-specific pooled effect, and the dashed lines represent the approximate 95% limits. The plot was used to explore small-study effects and potential publication bias within the network meta-analysis framework. BPD, bronchopulmonary dysplasia; OR, odds ratio

**Fig. 5:**
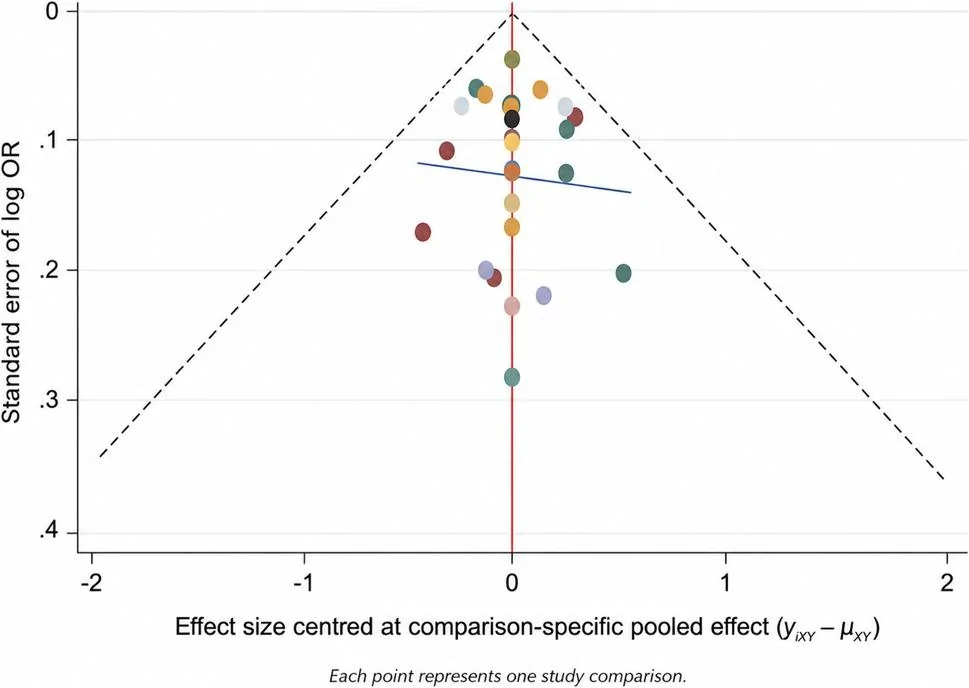
Comparison-adjusted funnel plot for pre-discharge mortality. Each point represents one study comparison centered at the comparison-specific pooled effect on the log OR scale. The vertical red line indicates the null-centered comparison-specific pooled effect, and the dashed lines represent the approximate 95% limits. The plot was used as an exploratory assessment of small-study effects and potential publication bias. Because the mortality network included sparse events and limited numbers of studies for several comparisons, visual interpretation should be cautious. OR, odds ratio

For BPD incidence, no clear asymmetry was observed (Fig. 4) in the comparison-adjusted funnel plot, and Egger’s test did not indicate statistically significant small-study effects (*P* = 0.14). For pre-discharge mortality (Fig. 5), Egger’s test yielded a P value of 0.09. However, given the relatively small number of studies and the sparse-event nature of mortality outcomes, both visual inspection and regression-based tests have limited statistical power and may yield unstable estimates. Therefore, the absence of statistically significant asymmetry should not be interpreted as definitive evidence of no publication bias, particularly for mortality.

### Sensitivity analysis (including Shennan1988 studies)

This study conducted sensitivity analyses based on different diagnostic criteria and birth weight. The results (Supplementary Material 2 Table S6) Sensitivity and subgroup analyses were performed to examine the robustness of the primary findings and to explore potential sources of heterogeneity, including BPD diagnostic definition, gestational age and/or birth weight category, and prophylactic versus therapeutic use. Overall, the direction of the estimates for BPD incidence was broadly consistent across several sensitivity analyses, although the magnitude and precision of effects varied across strata. For pre-discharge mortality, estimates were less stable and generally less precise, reflecting the smaller number of contributing studies and the sparse-event nature of this outcome.

These analyses should be interpreted as exploratory and hypothesis-generating. Several strata included limited numbers of studies, and subgroup classification was based on study-level rather than patient-level characteristics. In addition, differences in intervention type, timing, route of administration, baseline respiratory status, co-interventions, and clinical context may have contributed to heterogeneity. Therefore, these results should not be interpreted as causal subgroup effects or as evidence supporting subgroup-specific clinical recommendations.

### Meta-regression

Exploratory meta-regression was conducted to assess whether selected study-level characteristics were associated with variation in treatment effects. The examined covariates included BPD diagnostic definition, gestational age and/or birth weight category, treatment intent, and publication year where available. Overall, no covariate showed robust evidence of effect modification across outcomes. Variation in BPD diagnostic criteria appeared to contribute to between-study variability in BPD incidence estimates, but this finding should be interpreted cautiously.

The meta-regression analyses were limited by the number of available studies, incomplete reporting of some covariates, and the use of aggregate study-level rather than individual patient-level data. They were therefore intended to explore possible sources of heterogeneity rather than to establish causal effect modification. Detailed coefficients and 95% credible intervals are presented in (Supplementary Material 2 Table S7).

## Discussion

This network meta-analysis synthesized randomized evidence on pharmacologic strategies evaluated for reducing BPD incidence and pre-discharge mortality in preterm infants. In the BPD incidence network, azithromycin, budesonide, and caffeine showed favorable estimates compared with placebo. In the pre-discharge mortality network, hydrocortisone and nitric oxide plus vitamin A showed favorable estimates compared with placebo. However, these findings should be interpreted cautiously. The certainty of evidence was assessed at the outcome/network level rather than for each individual treatment comparison. The BPD incidence network was rated as having moderate certainty, whereas the pre-discharge mortality network was rated as having low certainty. Therefore, the observed treatment signals should be regarded as comparative and hypothesis-generating rather than definitive evidence of clinical superiority.

Several principles [58, 59] are important for interpreting this network meta-analysis. First, SUCRA values summarize the relative probability that an intervention ranks favorably within the network, but they do not establish clinically meaningful superiority. Rank order should not be interpreted independently of effect estimates, their 95% CrIs, network structure, and certainty of evidence. Second, some apparent comparative advantages in this network were informed partly by indirect comparisons, and precision was limited for several estimates, especially for sparsely connected treatments [60]. Third, not all included interventions represent broadly applicable routine preventive strategies. Some therapies are indication-specific and may reflect different clinical contexts, treatment goals, feasibility considerations, and baseline risk profiles [61]. Accordingly, the present findings are best understood as comparative signals within a shared preventive framework rather than as evidence that all included interventions are clinically interchangeable or suitable for routine use across neonatal settings.

Within the BPD incidence network, azithromycin and budesonide emerged among the more favorable interventions, while caffeine also showed a relatively stable signal. Previous literatures [62, 63] have suggested that azithromycin may be relevant in selected neonatal contexts because of its anti-inflammatory profile and its possible relationship to Ureaplasma colonization, whereas budesonide has been studied as an early anti-inflammatory strategy to reduce pulmonary injury, and caffeine has a more established role in neonatal respiratory care. However, these comparative findings should be interpreted within the corresponding certainty framework rather than on ranking results alone.

Within the pre-discharge mortality network, hydrocortisone and nitric oxide plus vitamin A were associated with lower odds of mortality than placebo. Prior studies have explored hydrocortisone as a potentially less harmful corticosteroid approach in extremely preterm infants, while nitric oxide- and vitamin A-related strategies have been discussed in selected respiratory or deficiency-related settings [64]. However, these signals should be interpreted with greater caution than the BPD incidence findings. Therefore, the apparently favorable findings for hydrocortisone and nitric oxide plus vitamin A should be regarded as exploratory comparative signals rather than definitive evidence supporting clinically meaningful superiority or routine use. SUCRA values were used only as probabilistic summaries of treatment ranking within each network. They were not used as stand-alone evidence of treatment superiority. This approach is particularly important for pre-discharge mortality, for which the certainty of evidence was low and several estimates were imprecise. Accordingly, the ranking results should be viewed as exploratory and should not be used alone to guide clinical decision-making.

The clinical implications of this study are therefore necessarily cautious. The present analysis provides comparative evidence rather than treatment recommendations. Given the heterogeneity of BPD definitions, variability in treatment timing and indication, limited long-term safety data, and the indirect nature of many comparisons, these findings should not be used in isolation to alter current guideline-based practice. Instead, they may help identify interventions that deserve further evaluation in carefully selected neonatal populations and may assist in prioritizing future comparative trials.

Several priorities for future research emerge from this network. First, more head-to-head randomized trials are needed in clinically coherent populations to reduce reliance on indirect evidence. Second, future studies should report BPD definitions, assessment timepoints, treatment timing, co-interventions, and baseline respiratory severity more consistently to improve interpretability across trials. Third, research should move beyond overall BPD incidence and focus more specifically on moderate-to-severe BPD, phenotype-informed subgroups, and competing-risk outcomes such as BPD or death. Finally, long-term respiratory morbidity and neurodevelopmental outcomes should be incorporated routinely, because short-term in-hospital outcomes alone cannot fully characterize the balance between benefit and harm for these therapies.

Several limitations should be considered. First, many comparisons were connected through placebo or standard care, and direct head-to-head evidence between active interventions was limited. Second, several treatment nodes were sparse, which reduced the precision of some network estimates and increased dependence on indirect evidence. Third, included interventions differed in mechanism, timing, route, indication, and clinical context, which may challenge the assumption of transitivity. Fourth, BPD definitions and assessment timepoints varied across trials, and death before BPD assessment may complicate interpretation of BPD incidence. Finally, long-term respiratory and neurodevelopmental outcomes were not consistently available. These limitations support a cautious interpretation of the findings as comparative signals rather than treatment recommendations.

The sensitivity analyses and meta-regression were designed to explore robustness and potential sources of heterogeneity rather than to support definitive subgroup conclusions. Although some subgroup estimates differed in magnitude, these differences should be interpreted cautiously because several strata included few studies, covariates were defined at the study level rather than the patient level, and reporting of potential effect modifiers was incomplete. Moreover, the included interventions varied substantially in mechanism, timing, dose, route, and clinical context. These factors limit the ability to attribute observed subgroup differences to a specific diagnostic definition, gestational age or birth weight category, or treatment intent. Accordingly, subgroup and meta-regression findings should be considered hypothesis-generating and should not be used to guide subgroup-specific treatment decisions.

## Conclusion

This network meta-analysis identified several comparative signals for pharmacologic strategies evaluated for BPD prevention and pre-discharge mortality in preterm infants. However, because several estimates were affected by indirect evidence, sparse nodes, and imprecision, and because the certainty of evidence was moderate for BPD incidence and low for pre-discharge mortality at the outcome/network level, these findings should be interpreted as evidence-mapping and hypothesis-generating rather than as treatment recommendations. Future studies should prioritize direct head-to-head comparisons, phenotype-or severity-specific analyses, and long-term respiratory and neurodevelopmental outcomes.

## Supplementary Information

Supplementary Material 1: Search Strategy

Supplementary Material 2: Figure S1: Risk of bias summary. Figure S2: Local inconsistency test for BPD. Figure S3: Local inconsistency test for pre-discharge mortality. Table S1. Table S2 Grade of evidence. Table S3 Data consistency Modeling (random model). Table S4 The league plots of main network meta-analysis. Table S5: The surface under the cumulative ranking curve of each treatment.Table S6 Sensitivity analysis results. Table S7 Meta-regression results.

## Data Availability

All data generated or analyzed during this study are included in this published article [and its supplementary information files].
