# Supplementary material for: Efficacy of pharmacologic therapies for reducing bronchopulmonary dysplasia and mortality in preterm infants: a network meta-analysis
Source: BMC Pediatr. 2026 Jul 28;26:782. doi: 10.1186/s12887-026-07408-y (PMC13504778; doi:10.1186/s12887-026-07408-y)
Supplement: Supplementary file 1 [file 12887_2026_7408_MOESM1_ESM.docx]

| Query | Results | Time |
| --- | --- | --- |
| (("Bronchopulmonary Dysplasia"[Mesh]) OR (((Bronchopulmonary Dysplasia[Title/Abstract]) OR (Dysplasia, Bronchopulmonary[Title/Abstract])) OR (BPD[Title/Abstract]))) AND (("prevention and control" [Subheading]) OR ((((((((("prevention and control"[Title/Abstract]) OR (prophylaxis[Title/Abstract])) OR (preventive therapy[Title/Abstract])) OR ("prevention and control"[Title/Abstract])) OR (preventive measures[Title/Abstract])) OR (prevention[Title/Abstract])) OR (control[Title/Abstract])) OR (decrease[Title/Abstract])) OR (reduce[Title/Abstract]))) | 509 | 22:01:52 |

Embase

| No. | Query | Results | Date |
| --- | --- | --- | --- |
| #18 | #17 AND 'randomized controlled trial'/de | 642 | 1-Sep-25 |
| #17 | #5 AND #16 | 8511 | 1-Sep-25 |
| #16 | #6 OR #7 OR #8 OR #9 OR #10 OR #11 OR #12 OR #13 OR #14 OR #15 | 8485447 | 1-Sep-25 |
| #15 | 'reduce':ab,ti | 1168589 | 1-Sep-25 |
| #14 | 'decrease':ab,ti | 1486167 | 1-Sep-25 |
| #13 | 'control':ab,ti | 4019774 | 1-Sep-25 |
| #12 | 'prevention':ab,ti | 877419 | 1-Sep-25 |
| #11 | 'preventive measures':ab,ti | 35612 | 1-Sep-25 |
| #10 | 'prevention and control':ab,ti | 27065 | 1-Sep-25 |
| #9 | 'preventive therapy':ab,ti | 5033 | 1-Sep-25 |
| #8 | 'prophylaxis':ab,ti | 174985 | 1-Sep-25 |
| #7 | 'prevention and control':ab,ti | 27065 | 1-Sep-25 |
| #6 | 'prevention and control'/exp | 2653934 | 1-Sep-25 |
| #5 | #1 OR #2 OR #3 OR #4 | 28020 | 1-Sep-25 |
| #4 | 'bpd':ab,ti | 17469 | 1-Sep-25 |
| #3 | 'dysplasia, bronchopulmonary':ab,ti | 10 | 1-Sep-25 |
| #2 | 'bronchopulmonary dysplasia':ab,ti | 12037 | 1-Sep-25 |
| #1 | 'lung dysplasia'/exp | 15462 | 1-Sep-25 |

Web of science

| # | Query | Results | Date |
| --- | --- | --- | --- |
| 1 | TS=(Bronchopulmonary Dysplasia) OR TS=(Dysplasia, Bronchopulmonary) OR TS=(BPD) | 21952 | 1-Sep-25 |
| 2 | TS=(Bronchopulmonary Dysplasia) OR TS=(Dysplasia, Bronchopulmonary) OR TS=(BPD) | 21952 | 1-Sep-25 |
| 3 | TS=(prevention and control) OR TS=(prophylaxis) OR TS=(preventive therapy) OR TS=(preventive measures) OR TS=(prevention) OR TS=(control) OR TS=(decrease) OR TS=(reduce) | 15531873 | 1-Sep-25 |
| 4 | #3 AND #2 | 10544 | 1-Sep-25 |
| 5 | TS=(randomized controlled trial) OR TS=(randomized) OR TS=(double-blind) OR TS=(placebo） | 1317005 | 1-Sep-25 |
| 6 | #5 AND #4 | 2226 | 1-Sep-25 |

Cochrane library

Date Run:	1-Sep-25

Comment:

ID	Search	Hits

#1	MeSH descriptor: [Bronchopulmonary Dysplasia] explode all trees	667

#2	(Bronchopulmonary Dysplasia):ti,ab,kw OR (Dysplasia, Bronchopulmonary):ti,ab,kw OR (BPD):ti,ab,kw	2857

#3	#1 or #2	2857

#4	(prevention and control):ti,ab,kw OR (prophylaxis):ti,ab,kw OR (preventive therapy):ti,ab,kw OR (preventive measures):ti,ab,kw OR (prevention):ti,ab,kw	226038

#5	(control):ti,ab,kw OR (decrease):ti,ab,kw OR (reduce):ti,ab,kw	735346

#6	#4 or #5	795898

#7	#3 and #6	1501
