# Supplementary material for: Efficacy of pharmacologic therapies for reducing bronchopulmonary dysplasia and mortality in preterm infants: a network meta-analysis
Source: BMC Pediatr. 2026 Jul 28;26:782. doi: 10.1186/s12887-026-07408-y (PMC13504778; doi:10.1186/s12887-026-07408-y)
Supplement: Supplementary file 2 [file 12887_2026_7408_MOESM2_ESM.docx]

Figure S1: Risk of bias summary

Figure S2: Local inconsistency test for BPD incidence. BPD: bronchopulmonary dysplasia, CrI: credible interval, VA: vitamin A, NO: nitric oxide.

Figure S3: Local inconsistency test for Pre-discharge mortality. CrI: credible interval, VA: vitamin A, NO: nitric oxide.

Table S1 The outcome distribution included in the article

| **Study** | **Included in overall review** | **Included in primary BPD network** | **Included in primary mortality network** | **Reason not included in primary BPD network** | **Reason not included in primary mortality network** |
| --- | --- | --- | --- | --- | --- |
| Russell 1995 | Yes | No | No | BPD defined using Shennan 1988; excluded from primary analysis and considered only in sensitivity analysis by BPD definition | Same as at left |
| Couser 1996 | Yes | No | No | BPD defined using Shennan 1988; excluded from primary analysis and considered only in sensitivity analysis by BPD definition | Same as at left |
| Denjean 1998 | Yes | No | No | BPD defined using Shennan 1988; excluded from primary analysis and considered only in sensitivity analysis by BPD definition | Same as at left |
| Cole 1999 | Yes | No | No | BPD defined using Shennan 1988; excluded from primary analysis and considered only in sensitivity analysis by BPD definition | Same as at left; pre-discharge mortality not reported in extractable form |
| Nupponen 2002 | Yes | No | No | BPD defined using Shennan 1988; excluded from primary analysis and considered only in sensitivity analysis by BPD definition | Same as at left |
| Ahola 2003 | Yes | No | No | BPD defined using Shennan 1988; excluded from primary analysis and considered only in sensitivity analysis by BPD definition | Same as at left |
| Davis 2003 | Yes | Yes | Yes | — | — |
| Ballard 2006 | Yes | Yes | Yes | — | — |
| Dani 2006 | Yes | No | No | BPD defined using Shennan 1988; excluded from primary analysis and considered only in sensitivity analysis by BPD definition | Same as at left |
| Doyle 2006 | Yes | No | No | BPD defined using Shennan 1988; excluded from primary analysis and considered only in sensitivity analysis by BPD definition | Same as at left |
| Kinsella 2006 | Yes | No | No | BPD defined using Shennan 1988; excluded from primary analysis and considered only in sensitivity analysis by BPD definition | Same as at left |
| Schmidt 2006 | Yes | Yes | Yes | — | — |
| Ballard 2007 | Yes | No | No | BPD defined using Shennan 1988; excluded from primary analysis and considered only in sensitivity analysis by BPD definition | Same as at left |
| Laughon 2009 | Yes | No | No | BPD defined using Shennan 1988; excluded from primary analysis and considered only in sensitivity analysis by BPD definition | Same as at left |
| Ballard 2011 | Yes | No | No | BPD defined using Shennan 1988; excluded from primary analysis and considered only in sensitivity analysis by BPD definition | Same as at left |
| Gharehbaghi 2012 | Yes | Yes | No | — | Pre-discharge mortality not reported in extractable form |
| Ng 2012 | Yes | Yes | No | — | Pre-discharge mortality not reported in extractable form |
| Terrin 2013 | Yes | Yes | No | — | Pre-discharge mortality not reported in extractable form |
| Gadhia 2014 | Yes | Yes | Yes | — | — |
| Kiatchoosakun 2014 | Yes | Yes | Yes | — | — |
| Bassler 2015 | Yes | Yes | Yes | — | — |
| Baud 2016 | Yes | Yes | Yes | — | — |
| Nakamura 2016 | Yes | Yes | Yes | — | — |
| Yeh 2016 | Yes | Yes | Yes | — | — |
| Hasan 2017 | Yes | Yes | Yes | — | — |
| Lin 2017 | Yes | Yes | Yes | — | — |
| Abounahia 2019 | Yes | Yes | Yes | — | — |
| Onland 2019 | Yes | Yes | Yes | — | — |
| Giridhar 2020 | Yes | Yes | Yes | — | — |
| Sung 2020 | Yes | Yes | Yes | — | — |
| Ahn 2021 | Yes | Yes | Yes | — | — |
| Dargaville 2021 | Yes | Yes | Yes | — | — |
| Gharehbaghi 2021 | Yes | Yes | Yes | — | — |
| Rysavy 2021 | Yes | Yes | Yes | — | — |
| Yao 2021 | Yes | Yes | No | — | Pre-discharge mortality not reported in extractable form |
| Aristizabal 2023 | Yes | Yes | No | — | Pre-discharge mortality not reported in extractable form |

Table S2 Grade of evidence

| **Outcome** | **Initial certainty** | **Risk of bias** | **Inconsistency / incoherence** | **Indirectness** | **Imprecision** | **Publication bias** | **Overall certainty** | **Main reasons for downgrading** | **Interpretation** |
| --- | --- | --- | --- | --- | --- | --- | --- | --- | --- |
| BPD incidence | High | Serious concerns | Not serious | Not serious / some concerns | Serious concerns | Not serious | Moderate | Downgraded mainly because some contributing trials had methodological concerns and several treatment comparisons, particularly sparse nodes, had wide 95% CrIs. No major statistical inconsistency was detected, but sparse network structure was considered in interpretation. | Moderate certainty. Favorable signals for azithromycin, budesonide, and caffeine should be interpreted as comparative evidence within the network, but not as definitive clinical superiority. |
| Pre-discharge mortality | High | Serious concerns | Not serious / some concerns | Some concerns | Serious concerns | Some concerns | Low | Downgraded because of methodological concerns, limited direct evidence for several comparisons, sparse mortality events, and wide 95% CrIs. Funnel-plot and regression-based assessments had limited power. | Low certainty. Favorable signals for hydrocortisone and nitric oxide plus vitamin A should be interpreted cautiously and considered hypothesis-generating. |

Table S3 Data consistency Modeling (random model)

| outcome | Consistency modeling | | Inconsistency modeling | |
| --- | --- | --- | --- | --- |
|  | I^2^ | DIC | I^2^ | DIC |
| BPD incidence | 3% | 94.6 | 5% | 95.2 |
| Predischarge mortality | 0% | 113.87 | 0% | 117.15 |

DIC: Deviance Information Criterion

**Supplementary Table S4A. League table for BPD incidence.**

| **Row vs column** | **Azithromycin** | **Budesonide** | **Caffeine** | **Erythromycin** | **Fluticasone** | **Hydrocortisone** | **Ibuprofen** | **Indomethacin** | **Nitric oxide** | **NO + vitamin A** | **Poractant alfa** | **Placebo** | **rhCuZnSOD** | **UCB-derived cells** | **Sildenafil** | **Vitamin A** | **Vitamin D** | **Zinc** |
| --- | --- | --- | --- | --- | --- | --- | --- | --- | --- | --- | --- | --- | --- | --- | --- | --- | --- | --- |
| **Azithromycin** | — |  |  |  |  |  |  |  |  |  |  |  |  |  |  |  |  |  |
| **Budesonide** | 0.48 (0.09, 1.82) | — |  |  |  |  |  |  |  |  |  |  |  |  |  |  |  |  |
| **Caffeine** | 0.38 (0.07, 1.42) | 0.79 (0.58, 1.07) | — |  |  |  |  |  |  |  |  |  |  |  |  |  |  |  |
| **Erythromycin** | 0.51 (0.06, 3.8) | 1.08 (0.27, 4.97) | 1.36 (0.35, 6.22) | — |  |  |  |  |  |  |  |  |  |  |  |  |  |  |
| **Fluticasone** | 0.32 (0.06, 1.33) | 0.67 (0.36, 1.23) | 0.85 (0.47, 1.53) | 0.62 (0.13, 2.71) | — |  |  |  |  |  |  |  |  |  |  |  |  |  |
| **Hydrocortisone** | 0.26 (0.05, 0.98) | 0.54 (0.37, 0.78) | 0.68 (0.48, 0.95) | 0.5 (0.11, 1.99) | 0.8 (0.43, 1.51) | — |  |  |  |  |  |  |  |  |  |  |  |  |
| **Ibuprofen** | 0.21 (0.04, 0.93) | 0.44 (0.22, 0.9) | 0.56 (0.28, 1.13) | 0.41 (0.08, 1.86) | 0.66 (0.27, 1.59) | 0.82 (0.39, 1.72) | — |  |  |  |  |  |  |  |  |  |  |  |
| **Indomethacin** | 0.16 (0.02, 0.81) | 0.33 (0.12, 0.89) | 0.41 (0.15, 1.12) | 0.3 (0.05, 1.61) | 0.49 (0.16, 1.51) | 0.61 (0.22, 1.69) | 0.74 (0.37, 1.49) | — |  |  |  |  |  |  |  |  |  |  |
| **Nitric oxide** | 0.29 (0.06, 1.1) | 0.61 (0.44, 0.83) | 0.77 (0.59, 1) | 0.56 (0.12, 2.2) | 0.91 (0.5, 1.65) | 1.13 (0.8, 1.61) | 1.38 (0.68, 2.78) | 1.85 (0.69, 4.99) | — |  |  |  |  |  |  |  |  |  |
| **NO + vitamin A** | 0.28 (0.05, 1.11) | 0.58 (0.36, 0.94) | 0.74 (0.47, 1.16) | 0.54 (0.11, 2.23) | 0.88 (0.43, 1.75) | 1.09 (0.66, 1.8) | 1.33 (0.6, 2.92) | 1.79 (0.62, 5.12) | 0.96 (0.63, 1.46) | — |  |  |  |  |  |  |  |  |
| **Poractant alfa** | 0.35 (0.07, 1.37) | 0.73 (0.46, 1.14) | 0.92 (0.61, 1.41) | 0.68 (0.14, 2.74) | 1.09 (0.55, 2.15) | 1.36 (0.84, 2.19) | 1.65 (0.76, 3.58) | 2.22 (0.78, 6.3) | 1.2 (0.78, 1.84) | 1.24 (0.71, 2.19) | — |  |  |  |  |  |  |  |
| **Placebo** | 0.25 (0.05, 0.93) | 0.52 (0.41, 0.66) | 0.66 (0.55, 0.79) | 0.49 (0.11, 1.88) | 0.78 (0.45, 1.37) | 0.97 (0.73, 1.3) | 1.18 (0.6, 2.33) | 1.59 (0.6, 4.2) | 0.86 (0.7, 1.05) | 0.89 (0.59, 1.35) | 0.72 (0.49, 1.05) | — |  |  |  |  |  |  |
| **rhCuZnSOD** | 0.27 (0.05, 1.12) | 0.57 (0.32, 1.02) | 0.72 (0.41, 1.27) | 0.53 (0.11, 2.27) | 0.85 (0.4, 1.85) | 1.06 (0.59, 1.95) | 1.3 (0.55, 3.05) | 1.74 (0.57, 5.25) | 0.94 (0.54, 1.66) | 0.98 (0.5, 1.92) | 0.79 (0.41, 1.5) | 1.09 (0.65, 1.87) | — |  |  |  |  |  |
| **UCB-derived cells** | 0.32 (0.05, 1.67) | 0.67 (0.25, 1.87) | 0.85 (0.32, 2.33) | 0.62 (0.1, 3.36) | 1 (0.33, 3.14) | 1.25 (0.45, 3.53) | 1.52 (0.46, 5.03) | 2.05 (0.52, 8.18) | 1.1 (0.41, 3.05) | 1.15 (0.4, 3.36) | 0.92 (0.32, 2.67) | 1.28 (0.49, 3.47) | 1.17 (0.39, 3.61) | — |  |  |  |  |
| **Sildenafil** | 0.25 (0.02, 2.5) | 0.52 (0.08, 3.49) | 0.66 (0.1, 4.41) | 0.48 (0.04, 4.88) | 0.78 (0.11, 5.57) | 0.97 (0.15, 6.57) | 1.18 (0.16, 8.73) | 1.59 (0.2, 13.14) | 0.86 (0.13, 5.74) | 0.89 (0.13, 6.15) | 0.72 (0.11, 4.92) | 1 (0.15, 6.6) | 0.91 (0.13, 6.48) | 0.78 (0.09, 6.51) | — |  |  |  |
| **Vitamin A** | 0.31 (0.06, 1.2) | 0.65 (0.44, 0.97) | 0.82 (0.57, 1.18) | 0.6 (0.13, 2.42) | 0.97 (0.51, 1.85) | 1.21 (0.79, 1.85) | 1.47 (0.7, 3.1) | 1.98 (0.71, 5.49) | 1.07 (0.76, 1.51) | 1.11 (0.69, 1.79) | 0.89 (0.54, 1.46) | 1.24 (0.91, 1.71) | 1.14 (0.61, 2.1) | 0.97 (0.34, 2.69) | 1.25 (0.18, 8.26) | — |  |  |
| **Vitamin D** | 0.12 (0.02, 0.59) | 0.25 (0.1, 0.62) | 0.32 (0.12, 0.77) | 0.23 (0.04, 1.16) | 0.37 (0.13, 1.06) | 0.47 (0.18, 1.17) | 0.57 (0.18, 1.72) | 0.76 (0.2, 2.83) | 0.41 (0.16, 1.01) | 0.43 (0.16, 1.13) | 0.34 (0.13, 0.89) | 0.48 (0.19, 1.15) | 0.44 (0.15, 1.22) | 0.37 (0.1, 1.39) | 0.48 (0.06, 3.74) | 0.38 (0.15, 0.98) | — |  |
| **Zinc** | 0.41 (0.07, 2) | 0.85 (0.35, 2.13) | 1.08 (0.45, 2.67) | 0.79 (0.14, 3.97) | 1.27 (0.46, 3.64) | 1.58 (0.65, 4.03) | 1.93 (0.65, 5.87) | 2.6 (0.71, 9.65) | 1.4 (0.58, 3.48) | 1.45 (0.56, 3.88) | 1.17 (0.46, 3.07) | 1.63 (0.69, 3.96) | 1.49 (0.54, 4.17) | 1.27 (0.34, 4.73) | 1.64 (0.21, 12.76) | 1.31 (0.53, 3.36) | 3.41 (1, 12.26) | — |

Comparisons should be read as column treatment versus row treatment. Effect estimates are presented as odds ratios (ORs) with 95% credible intervals (CrIs). ORs < 1 favor the column treatment, whereas ORs > 1 favor the row treatment. Only the lower triangle is populated to avoid duplication. All treatment labels are standardized generic names or clearly defined generic short labels. BPD, bronchopulmonary dysplasia; OR, odds ratio; CrI, credible interval.

**Supplementary Table S4B. League table for pre-discharge mortality.**

| **Row vs column** | **Budesonide** | **Caffeine** | **Fluticasone** | **Hydrocortisone** | **Ibuprofen** | **Indomethacin** | **Nitric oxide** | **NO + vitamin A** | **Poractant alfa** | **Placebo** | **rhCuZnSOD** | **UCB-derived cells** | **Sildenafil** | **Vitamin A** |
| --- | --- | --- | --- | --- | --- | --- | --- | --- | --- | --- | --- | --- | --- | --- |
| **Budesonide** | — | 0.86 (0.52, 1.42) | 1.33 (0.47, 4.06) | 0.64 (0.4, 1) | 0.96 (0.27, 3.44) | 0.84 (0.19, 3.87) | 0.88 (0.56, 1.39) | 0.47 (0.23, 0.95) | 1.21 (0.6, 2.47) | 0.92 (0.67, 1.26) | 0.88 (0.32, 2.47) | 3.59 (0.37, 95.95) | 0.37 (0.04, 2.38) | 0.85 (0.48, 1.48) |
| **Caffeine** |  | — | 1.55 (0.53, 4.86) | 0.74 (0.44, 1.24) | 1.11 (0.31, 4.1) | 0.98 (0.21, 4.58) | 1.03 (0.62, 1.71) | 0.55 (0.25, 1.15) | 1.4 (0.67, 2.99) | 1.07 (0.72, 1.59) | 1.03 (0.36, 2.96) | 4.18 (0.42, 113.21) | 0.43 (0.05, 2.84) | 0.99 (0.54, 1.82) |
| **Fluticasone** |  |  | — | 0.48 (0.16, 1.38) | 0.72 (0.14, 3.54) | 0.63 (0.1, 3.81) | 0.66 (0.22, 1.91) | 0.35 (0.1, 1.17) | 0.91 (0.26, 2.99) | 0.69 (0.24, 1.88) | 0.66 (0.15, 2.68) | 2.72 (0.22, 82.71) | 0.27 (0.02, 2.26) | 0.64 (0.2, 1.93) |
| **Hydrocortisone** |  |  |  | — | 1.5 (0.42, 5.42) | 1.32 (0.29, 6.06) | 1.38 (0.87, 2.21) | 0.74 (0.35, 1.5) | 1.9 (0.93, 3.91) | 1.44 (1.04, 2.01) | 1.38 (0.49, 3.89) | 5.64 (0.57, 151.77) | 0.58 (0.06, 3.75) | 1.34 (0.75, 2.35) |
| **Ibuprofen** |  |  |  |  | — | 0.88 (0.4, 1.99) | 0.92 (0.26, 3.33) | 0.49 (0.12, 1.98) | 1.27 (0.31, 5.08) | 0.96 (0.28, 3.29) | 0.92 (0.19, 4.43) | 3.83 (0.28, 118.26) | 0.38 (0.03, 3.55) | 0.89 (0.24, 3.32) |
| **Indomethacin** |  |  |  |  |  | — | 1.05 (0.23, 4.74) | 0.56 (0.11, 2.76) | 1.43 (0.28, 7.09) | 1.09 (0.25, 4.73) | 1.04 (0.18, 6.13) | 4.35 (0.28, 148.07) | 0.43 (0.03, 4.63) | 1.01 (0.21, 4.71) |
| **Nitric oxide** |  |  |  |  |  |  | — | 0.54 (0.27, 1) | 1.37 (0.67, 2.81) | 1.04 (0.75, 1.44) | 1 (0.35, 2.81) | 4.05 (0.41, 109.9) | 0.42 (0.05, 2.69) | 0.97 (0.58, 1.58) |
| **NO + vitamin A** |  |  |  |  |  |  |  | — | 2.55 (1.05, 6.45) | 1.93 (1.05, 3.81) | 1.86 (0.58, 6.09) | 7.65 (0.72, 213) | 0.78 (0.08, 5.56) | 1.79 (0.88, 3.78) |
| **Poractant alfa** |  |  |  |  |  |  |  |  | — | 0.76 (0.4, 1.43) | 0.73 (0.23, 2.36) | 2.99 (0.28, 82.68) | 0.31 (0.03, 2.12) | 0.7 (0.32, 1.54) |
| **Placebo** |  |  |  |  |  |  |  |  |  | — | 0.96 (0.36, 2.56) | 3.9 (0.41, 104.25) | 0.4 (0.05, 2.53) | 0.93 (0.58, 1.47) |
| **rhCuZnSOD** |  |  |  |  |  |  |  |  |  |  | — | 4.11 (0.34, 123.4) | 0.42 (0.04, 3.38) | 0.96 (0.33, 2.85) |
| **UCB-derived cells** |  |  |  |  |  |  |  |  |  |  |  | — | 0.1 (0, 1.94) | 0.24 (0.01, 2.39) |
| **Sildenafil** |  |  |  |  |  |  |  |  |  |  |  |  | — | 2.3 (0.35, 21.08) |
| **Vitamin A** |  |  |  |  |  |  |  |  |  |  |  |  |  | — |
| **Vitamin D** |  |  |  |  |  |  |  |  |  |  |  |  |  |  |
| **Zinc** |  |  |  |  |  |  |  |  |  |  |  |  |  |  |

Comparisons should be read as column treatment versus row treatment. Effect estimates are presented as odds ratios (ORs) with 95% credible intervals (CrIs). ORs < 1 favor the column treatment, whereas ORs > 1 favor the row treatment. Only the upper triangle is populated to avoid duplication. All treatment labels are standardized generic names or clearly defined generic short labels. OR, odds ratio; CrI, credible interval.

**Generic short-label definitions:** Azithromycin; Budesonide; Caffeine; Erythromycin; Fluticasone, fluticasone propionate; Hydrocortisone; Ibuprofen; Indomethacin; Nitric oxide; NO + vitamin A, nitric oxide plus vitamin A; Poractant alfa; Placebo; rhCuZnSOD, recombinant human CuZn superoxide dismutase; UCB-derived cells, UCB-derived stem cells; Sildenafil; Vitamin A; Vitamin D; Zinc.

Table S5: The surface under the cumulative ranking curve of each treatment.

| Pharmacologic intervention | BPD incidence | Pre-discharge mortality |
| --- | --- | --- |
| azithromycin | 0.93 | / |
| budesonide | 0.85 | 0.38 |
| caffeine | 0.71 | 0.52 |
| erythromycin | 0.74 | / |
| fluticasone propionate | 0.54 | 0.27 |
| hydrocortisone | 0.34 | 0.77 |
| ibuprofen | 0.26 | 0.45 |
| indomethacin | 0.15 | 0.53 |
| nitric oxide | 0.47 | 0.5 |
| nitric oxide plus vitamin A | 0.43 | 0.87 |
| poractant alfa | 0.63 | 0.28 |
| placebo | 0.3 | 0.46 |
| recombinant human CuZn superoxide dismutase | 0.41 | 0.5 |
| UCB-derived stem cells | 0.53 | 0.13 |
| sildenafil | 0.42 | 0.81 |
| vitamin A | 0.52 | 0.53 |
| vitamin D | 0.07 | / |
| zinc | 0.68 | / |

SUCRA values should not be interpreted as evidence of clinical superiority or treatment recommendation.

Table S6 Sensitivity analysis results

| outcomes | subgroup | Number of studies | Heterogeneity (%) | OR 95% CrI |
| --- | --- | --- | --- | --- |
| BPD incidence | Shennan 1988 definition | 14 | 78.6 | 0.65(0.41, 0.99) |
|  | NIH 2001 definition | 16 | 28.5 | 0.77(0.67, 0.89) |
|  | NIH 2018 definition | 9 | 47.1 | 0.84(0.64, 1.10) |
|  | GA <28 weeks and/or BW <1000 | 31 | 32.7 | 0.85(0.75, 0.95) |
|  | GA >28 weeks and/or BW >1000 | 11 | 74 | 0.4(0.23, 0.69) |
|  | Prophylacti | 21 | 67.3 | 0.84(0.65, 0.91) |
|  | Treatment | 21 | 77.1 | 0.77 (0.53, 0.89) |
| Pre-discharge mortality | Shennan 1988 definition | 12 | 9.3 | 1.01(0.8, 1.27) |
|  | NIH 2001 definition | 16 | 3.0 | 0.95(0.8, 1.12) |
|  | NIH 2018 definition | 6 | 0 | 0.79(0.61, 1.05) |
|  | GA <28 weeks and/or BW <1000 | 29 | 0 | 0.90(0.79, 1.02) |
|  | GA >28 weeks and/or BW >1000 | 5 | 0 | 1.50(0.94, 2.38) |
|  | Prophylacti | 14 | 7.3 | 0.64(0.35, 0.84) |
|  | Treatment | 10 | 22.1 | 0.82 (0.70, 0.92) |

Effect estimates are presented as odds ratios (ORs) with 95% credible intervals (CrIs). These analyses were exploratory and were conducted to examine the robustness of the primary findings and to explore potential sources of heterogeneity. Subgroup classifications were based on study-level characteristics rather than individual patient-level data. Several strata included limited numbers of studies, and estimates should not be interpreted as causal subgroup effects or as evidence supporting subgroup-specific clinical recommendations. BPD, bronchopulmonary dysplasia; CrI, credible interval; OR, odds ratio.

Table S7 Meta-regression results

| Outcomes | Year | | Diagnostic criteria for BPD | | Treatment | | GA and/or BW | |
| --- | --- | --- | --- | --- | --- | --- | --- | --- |
|  | coefficients | P | coefficients | P | coefficients | P | coefficients | P |
| BPD incidence | -0.12 | 0.13 | 0.54 | 0.02 | -0.82 | 0.45 | 0.52 | 0.78 |
| Predischarge mortality | 0.75 | 0.77 | 0.69 | 0.10 | -0.19 | 0.39 | -0.25 | 0.16 |

Supplementary Methods: Bayesian network meta-analysis settings and reproducibility details

Model specification and prior distributions

The Bayesian network meta-analysis was performed using R version 4.2.3 and the gemtc package. Binary outcomes were modeled using a binomial likelihood with a logit link function. Treatment effects were summarized as odds ratios (ORs) with 95% credible intervals (CrIs). A random-effects consistency model was used for the primary analysis, and an inconsistency model was fitted for comparison of model fit using the deviance information criterion (DIC).

For the basic treatment-effect parameters, vague normal prior distributions were used on the log-odds scale:

d ~ Normal(0, 10,000)

where *d* denotes the relative treatment effect. For the between-study heterogeneity standard deviation, a vague uniform prior distribution was used:

τ ~ Uniform(0, 5)

where *τ* denotes the between-study standard deviation on the log-odds scale. The same prior distributions were used for both the BPD incidence network and the pre-discharge mortality network.

Handling of zero-event studies

Zero-event patterns were assessed separately for BPD incidence and pre-discharge mortality. For each outcome-specific network, we identified trials with zero events in one treatment arm and trials with zero events in all treatment arms.

Trials with zero events in one arm were retained in the Bayesian network meta-analysis. When required for model estimation and computational stability, a continuity correction of 0.5 was added to all cells of the corresponding study-level two-by-two table. This correction was applied only to studies with at least one zero-event arm and was applied consistently across all arms within the affected study. Trials with zero events in all arms do not contribute information to relative treatment effects for binary outcomes; therefore, if present, they were retained in the systematic review and outcome-availability table but excluded from the corresponding quantitative synthesis.

For BPD incidence, [insert number] one-arm zero-event trial(s) and [insert number] all-arm zero-event trial(s) were identified. For pre-discharge mortality, [insert number] one-arm zero-event trial(s) and [insert number] all-arm zero-event trial(s) were identified. The handling strategy is summarized in Supplementary Table S7.

MCMC settings and convergence diagnostics

For each outcome-specific network, four Markov chain Monte Carlo chains were run. Each chain included 1,000 burn-in/adaptation iterations followed by 2,000 sampling iterations, with a thinning interval of 1. Therefore, 4,000 post-burn-in samples were retained for posterior inference in each outcome-specific network.

Convergence was assessed using the Gelman–Rubin diagnostic, potential scale reduction factor (PSRF), visual inspection of trace plots, and effective sample size. Across monitored parameters, PSRF values ranged from 1.00–1.03 for the BPD incidence network and from 1.00–1.05 for the pre-discharge mortality network. Trace plots showed adequate mixing of the four chains without evidence of systematic drift. Effective sample sizes were reviewed for treatment-effect parameters and heterogeneity parameters. The effective sample sizes were generally sufficient for posterior inference, although some parameters related to sparsely connected treatment nodes, particularly in the pre-discharge mortality network, had relatively lower effective sample sizes. These diagnostic results are summarized in Supplementary Table S8.

| Outcome | One-arm zero-event trials | All-arm zero-event trials | Continuity correction | Handling strategy |
| --- | --- | --- | --- | --- |
| BPD incidence | 0 | 0 | Not required | No zero-event trial was identified in the primary BPD incidence network; therefore, no continuity correction was required. |
| Pre-discharge mortality | 4 | 1 | 0.5 | One-arm zero-event trials were retained with a 0.5 continuity correction when required. The all-arm zero-event trial was retained in the systematic review but excluded from the mortality network because it did not contribute information to relative treatment effects. |

| Item | BPD incidence network | Pre-discharge mortality network |
| --- | --- | --- |
| Software | R version 4.2.3 | R version 4.2.3 |
| Package | gemtc | gemtc |
| Model | Bayesian random-effects NMA | Bayesian random-effects NMA |
| Likelihood | Binomial | Binomial |
| Link function | Logit | Logit |
| Effect measure | OR with 95% CrI | OR with 95% CrI |
| Primary model | Consistency model | Consistency model |
| Number of chains | 4 | 4 |
| Burn-in/adaptation iterations | 1,000 per chain | 1,000 per chain |
| Sampling iterations | 2,000 per chain | 2,000 per chain |
| Thinning interval | 1 | 1 |
| Total post-burn-in samples | 4,000 | 4,000 |
| Prior for treatment effects | Normal(0, 10,000) | Normal(0, 10,000) |
| Prior for heterogeneity SD | Uniform(0, 5) | Uniform(0, 5) |
| PSRF range | 1.00–1.03 | 1.00–1.05 |
| Effective sample size summary | Most monitored parameters had ESS >1,000; minimum ESS = 1 | Most monitored parameters had ESS >800; lower ESS was observed for sparse treatment nodes; minimum ESS = 1 |
